# FvBck1, a component of cell wall integrity MAP kinase pathway, is required for virulence and oxidative stress response in sugarcane Pokkah Boeng pathogen

**DOI:** 10.3389/fmicb.2015.01096

**Published:** 2015-10-08

**Authors:** Chengkang Zhang, Jianqiang Wang, Hong Tao, Xie Dang, Yang Wang, Miaoping Chen, Zhenzhen Zhai, Wenying Yu, Liping Xu, Won-Bo Shim, Guodong Lu, Zonghua Wang

**Affiliations:** ^1^Fujian Province Key Laboratory of Pathogenic Fungi and Mycotoxins, College of Life Sciences, Fujian Agriculture and Forestry UniversityFuzhou, China; ^2^Fujian University Key Laboratory for Functional Genomics of Plant Fungal Pathogens, Fujian Agriculture and Forestry UniversityFuzhou, China; ^3^Key Laboratory of Sugarcane Biology and Genetic Breeding, Ministry of Agriculture, Fujian Agriculture and Forestry UniversityFuzhou, China; ^4^Department of Plant Pathology and Microbiology, Texas A&M UniversityCollege Station, TX, USA

**Keywords:** *Fusarium verticillioides*, Pokkah Boeng, MAP kinase pathway, osmotic stress, superoxide scavenging, pathogenicity, fumonisin B1

## Abstract

*Fusarium verticillioides* (formerly *F. moniliforme*) is suggested as one of the causal agents of Pokkah Boeng, a serious disease of sugarcane worldwide. Currently, detailed molecular and physiological mechanism of pathogenesis is unknown. In this study, we focused on cell wall integrity MAPK pathway as one of the potential signaling mechanisms associated with Pokkah Boeng pathogenesis. We identified *FvBCK1* gene that encodes a MAP kinase kinase kinase homolog and determined that it is not only required for growth, micro- and macro-conidia production, and cell wall integrity but also for response to osmotic and oxidative stresses. The deletion of *FvBCK1* caused a significant reduction in virulence and FB1 production, a possibly carcinogenic mycotoxin produced by the fungus. Moreover, we found the expression levels of three genes, which are known to be involved in superoxide scavenging, were down regulated in the mutant. We hypothesized that the loss of superoxide scavenging capacity was one of the reasons for reduced virulence, but overexpression of catalase or peroxidase gene failed to restore the virulence defect in the deletion mutant. When we introduced *Magnaporthe oryzae MCK1* into the FvBck1 deletion mutant, while certain phenotypes were restored, the complemented strain failed to gain full virulence. In summary, FvBck1 plays a diverse role in *F. verticillioides*, and detailed investigation of downstream signaling pathways will lead to a better understanding of how this MAPK pathway regulates Pokkah Boeng on sugarcane.

## Introduction

Sugarcane is an important feedstock mainly used for sugar production in many countries such as Brazil, India, Iran, and China (Wu et al., 2006; Golabi et al., 2009; Vishwakarma et al., 2013). It is also an important bioenergy crop heavily utilized in Brazil (Luo et al., 2009). Pokkah Boeng is one of the most serious sugarcane diseases, and occurs in most sugarcane fields all over the world including China (Rott, 2000; Wu et al., 2006; Lin et al., 2014). Historically, the pathogen was known as *Fusarium moniliforme* Sheldon (teleomorph *Gibberella fujikuroi* complex), but recent advanced taxonomical studies suggest that the pathogen can be recognized as *F. verticillioides, F. proliferatum*, or *F. fujikuroi*. Early in 1989, Pokkah Boeng outbreak was reported in sugarcane fields in Guangdong province that led to huge economic losses (Huang, 1993), and similar disease epidemic appeared again in Guangxi province in 2005 (Wu et al., 2006). While there is no recent epidemic reported in China, Pokkah Boeng is still considered a serious and an emerging risk for sugarcane production. However, due to the complexity of the disease, little research has been done to better understand the mechanism of pathogenesis particularly at the molecular level.

*F. verticillioides* (Sacc.) Nirenberg (formerly known as *F. moniliforme*, teleomorph *Gibberella moniliformis* Wineland, formerly *Gibberella fujiukuroi* population A) is a widely distributed pathogen of important agronomic field crops worldwide. The fungus is a notorious pathogen of maize, causing kernel or ear rot, stalk rot, root rot, and seedling blight (Kommedahl and Windels, 1981), and is commonly transmitted through seedborne infection (Wilke et al., 2007). The fungus produces two distinct forms of asexual conidia, i.e., oval, hyaline, and mostly single-celled microconidia and multi-celled, canoe-shaped macroconidia, that allows effective dissemination. In addition to causing diseases, *F. verticillioides* produces mycotoxins on infested grains, notably fumonisin B1 (FB1), which has neurotoxic, immunotoxic, nephrotoxic, hepatotoxic, and potential carcinogenic properties in humans and animals (Stockmann-Juvala and Savolainen, 2008). The gene cluster (*FUM* cluster) responsible for the biosynthesis of FB1 has been characterized (Brown et al., 2007). However, the precise mechanism of how *F. verticillioides* regulates the *FUM* cluster to produce FB1 is not completely understood (Woloshuk and Shim, 2013).

Fungi are commonly found in diverse environments, withstanding an adverse array of stresses and challenges. Cell wall is a critical element of fungi that helps withstand environmental adversities such as high temperature, saline-alkali soil, osmotic, and oxidative stresses. To perceive a diverse array of extracellular signals, fungi utilize highly conserved mitogen-activated protein kinase (MAPK) signaling pathways, which were extensively investigated in *Saccharomyces cerevisiae* and other fungi (Seger and Krebs, 1995; Chen and Thorner, 2007; Wang et al., 2011; Li et al., 2012; Krijgsheld et al., 2013). Five MAP kinase pathways, each with distinct functions, are known in fungi (Chen and Thorner, 2007), and of these the Slt2/Mpk1-dependent response is known as the cell wall integrity (CWI) MAP kinase pathway (Fuchs and Mylonakis, 2009).

In *S. cerevisiae*, the Slt2/Mpk1 pathway is activated by protein kinase C1 (PKC1), which is activated by a small GTPase Rho1. PKC1 then goes on to activate the Slt2/Mpk1 cell wall integrity pathway composed of the MAPKKK (Bck1), the MAPKKs (Mkk1 and Mkk2), and the MAPK (Slt2/Mpk1) cascade (Levin, 2005). The role of these orthologous kinases has been studied in a number of fungal pathogens. For instance, *Aspergillus fumigatus* Bck1 ortholog AfBck1 controls cell wall signaling, pyomelanin formation, and stress response (Valiante et al., 2009). Disruption of *Magnaporthe oryzae* Bck1 ortholog Mck1 led to autolysis and hypersensitivity against cell wall-degrading enzyme, and failed to grow *in planta* (Jeon et al., 2008). Yeast Slt2 ortholog Mgv1 plays multiple important roles in sexual reproduction, plant infection, and cell wall integrity in *F. graminearum* (Hou et al., 2002). In *M. oryzae*, Mps1 is indispensable for cell wall integrity, conidiation, and plant infection (Xu et al., 1998).

A number of nuclear targets are regulated by Slt2/Mpk1, including the SBF complex acting as a transcriptional activator of cell cycle-dependent genes (Nasmyth and Dirick, 1991). Slt2/Mpk1 also activates the MADS-box transcription factor Rlm1, which regulates the expression of at least 25 genes in *S. cerevisiae*, most of which have been implicated in cell wall biogenesis and function (Jung et al., 2002). Rlm1 also regulates serine/threonine protein phosphatases Ppz1 and Ppz2, which are important for K^+^ and pH homeostasis, salt tolerance, cell wall integrity, and cell cycle progression (Yenush et al., 2002).

As described earlier, CWI signaling pathway plays diverse, but also critical, roles in the physiology and stress response in fungi. While the role of CWI pathway and its components in *F. verticillioides* are not clearly defined to date, we anticipate that this pathway is important for growth and stress response, particularly those originating from the host. We also hypothesized that CWI pathway is involved in *F. verticillioides* virulence. Notably, we selected *F. verticillioides* as a mean to study Pokkah Boeng pathogenesis thanks to its molecular genetic resources and tools (Xu and Leslie, 1996; Brown et al., 2005; Ma et al., 2010). In this study, we identified *S. cerevisiae* Bck1 homolog in *F. verticillioides*, a MAPKKK designated FvBck1, and characterized its role in sugarcane pathogenesis. Since FvBck1 is the most upstream component of the CWI MAPK cascade, we also investigated its impact on downstream signaling components and on osmotic and oxidative stress responses.

## Materials and methods

### Fungal strains and growth conditions

*F. verticillioides* wild-type strain 7600 (Choi and Xu, 2010) and other derivative strains (ΔFvbck1, ΔFvbck1-8, FvBck1-C, FvBck1-C2, CAT-OE13, POX-OE6, MoC-3, and MoC-6) described in this study were stored in 20% (v/v) glycerin at −80°C. For vegetative growth assays, all strains were grown on/in the sugarcane juice agar medium (SJA: sugarcane juice from boiled and filtered 500 g sugarcane, and 20 g agar per 1 L) or complete medium [CM: 6 g yeast extract, 6 g casein hydrolysate, 10 g sucrose, and 20 g agar (for solid medium) per 1L] at 26°C. Hyphae from liquid CM were stained with 5 g/ml DAPI for nuclei observation (Seong et al., 2008). For conidiation assays, all strains were grown in/on mung bean juice medium (MBJ: mung bean juice from boiled and filtered 40 g mung bean per 1 L) and solid modified Bilai's medium (1 g KH_2_PO_4_, 1 g KNO_3_, 0.5 g KCl, 0.5 g MgSO_4_, 0.2 g starch, 0.2 g glucose and 15 g agar per 1 L) (Joffe, 1963). To test the sensitivity against osmotic regulators, H_2_O_2_ or cell-wall-disrupting agents, vegetative growth was assayed on CM plates or in liquid CM with sucrose, sorbitol, KCl, NaCl, H_2_O_2_, calcofluor white (CFW), or Congo red (CR) (with concentration described in Results) at 26°C. To test the sensitivity against high temperature, all strains were grown on CM plates at 33°C. Microscopy observation was performed on an Olympus BX51 Microscope. All experiments were repeated at least three times.

### Gene replacement, complementation, and overexpression

To replace *FvBCK1* gene in *F. verticillioides*, a 1.2-kb fragment upstream of the *FvBCK1* in the genome was amplified with primer pair 1F/1R (Supplementary Image 1A, Table S1) and cloned into the *Kpn*I and *Hin*dIII sites on pCX62 (Zhao et al., 2004), and the new construct was named pBCK11. Then a 1.1-kb fragment downstream of *FvBCK1* was amplified with primers pair 2F/2R (Supplementary Image 1A, Table S1) and cloned between the *Spe*I and *Xba*I sites in pBCK11. The resulting construct was the *FvBCK1* gene replacement vector pBCK1, which had a hygromycin phosphotransferase gene (*HPH*) as the selectable marker flanked by the *FvBCK1* upstream and downstream sequences. The fragment, amplified with primer pair 3F/3R (Supplementary Image 1A, Table S1), was then transformed into protoplasts of the WT strain 7600. Hygromycin-resistant transformants were screened by PCR with primer pair 4F/4R or 5F/HR (Supplementary Image 1A, Table S1) to confirm whether *FvBCK1* gene was replaced with *HPH* gene. The complementation was performed by co-transforming pKNT vector and a 7.55-kb PCR amplicon containing the native promoter and *FvBCK1* gene (using primer pair 5F/5R) or a 8.98-kb amplicon with the native promoter and *MCK1* gene (using primer pair MoC1F/MoC1R) (Supplementary Image 1A, Table S1) into ΔFvbck1 strain. Complemented strains were obtained by screening for neomycin-resistant transformants and by subsequent PCR with primer pair 4F/4R or MoC1F/MoC1R (Supplementary Image 1A, Table S1). For gene overexpression, we fused the promoter of RP27 [amplified with primer pairs RP27F/RP27R-CAT or RP27F/RP27R-POX from plasmid pSM565 (GenBank AY142483)] with ORF of *CAT* (amplified with CAT-OEF/CAT-OER primers) or *POX* (amplified with POX-OEF/POX-OER primers) by joint-PCR strategy (Table S1). Then we transformed the product fragment with pKNT into the ΔFvbck1 mutant strain. The neomycin-resistant transformants were confirmed by PCR and qRT-PCR with primer pair QF-CAT/QR-CAT or QF-POX/QR-POX (Table S1). All the mutant strains were further confirmed by Southern blot analysis and/or RT-PCR.

### Southern blot analysis

For Southern blot analysis, genomic DNA isolated from WT, ΔFvbck1 and FvBck1-C strains were digested with *Kpn*I, separated by electrophoresis on 1% agarose gels and transferred onto a Hybond N+ membrane (Amersham Pharmacia Biotech, Piscathaway, New Jersey, USA). The probe was amplified with primers PF/PR (Supplementary Image 1A, Table S1). According to the manufacturer's instructions, the verification was done using DIG-High Prime DNA Labeling and Detection Starter Kit (Roche, Mannheim, Germany).

### RNA extraction and quantitative real-time PCR

Total RNA of each strain was extracted from the infected sugarcanes (including the autoclaved sugarcanes) after 7-day incubation or liquid CM media after 3-day incubation using TRNzol reagent according to the manufacturer's instructions (Tiangen Biotech, Beijing, China). First strand cDNA was synthesized with the FastQuant RT Kit (Tiangen Biotech) following the manufacturer's instructions. For quantitative real-time PCR, *FV12888, FV11221*, and *FV01940* genes were amplified with corresponding pair of primers (Table S1) and quantified with SuperReal PreMix Plus (SYBR Green) (Tiangen Biotech). The beta tubulin gene *FV04081* was used as an endogenous control. The relative quantification of the transcripts was calculated by the 2^−ΔΔ*Ct*^ method (Livak and Schmittgen, 2001). All qRT-PCR assays were conducted in technical triplicates for each sample, and the experiment was repeated at least three times.

### Pathogenicity assays and fumonisin B1 analysis

For fungal infection of sugarcane (cultivar Heiganzhe from Guangxi Zhuang Autonomous Region of China), conidia were harvested from 3-day-old CM cultures and resuspended in sterile distilled water to 2 × 10^7^ conidia/ml. Inter-nodal regions of the sugarcane stalk (including the autoclaved sugarcane as control) were punctured (1.5–2.0 cm deep) with sterile needle and a 50-μl conidial suspension was inoculated into the wound by microsyringe (Supplementary Image 2). After 7 days of incubation at 25°C temperature, stalks were split open longitudinally and the disease symptoms were photographed (Supplementary Image 2). Infection of corn stalks (cultivar Pioneer 2375) was performed following the protocol previously described (Sagaram and Shim, 2007). For FB1 extraction assay, corn kernels were prepared and inoculated as described by Bluhm and Woloshuk (2005). Subsequent FB1 extraction and analysis was performed following the method described by Kim and Woloshuk (2008). Fungal DNA level was measured by qRT-PCR to quantify fungal biomass in the inoculated corn kernels and was used to normalize FB1 levels in kernel samples.

## Results

### Identification of the Bck1 homolog in *F. verticillioides*

MAPK networks are one of the most important signaling pathways in eukaryotic organisms. Here, we selected a homolog of Bck1, the MAPKK kinase in the CWI pathway, to investigate how this signaling pathway impacts the physiology and virulence of *F. verticillioides*. BLAST analyses of *F. verticillioides* genome using *S. cerevisiae* Bck1 (Lee and Levin, 1992) and *M. oryzae* Mck1 (Jeon et al., 2008) as queries resulted in the identification of FVEG_05000 locus encoding a putative Bck1 homolog, which we designated herein as FvBck1 (*F**usarium* *v**erticillioides* Bck1). FvBck1 is predicted to contain a conserved serine/threonine protein kinase catalytic domain at its C-terminus for protein phosphorylation (http://www.broadinstitute.org/annotation/genome/fusarium_group/MultiHome.html). Based on the identity of amino acid sequences amongst homologs, namely those found in *S. cerevisiae, A. nidulans, Neurospora crassa, M. oryzae*, and *F. graminearum*, FvBck1 can be classified in the Bck1 MAPKK kinase family (Supplementary Image 3).

### Generation of the *FvBCK1* deletion mutants and complementation strains

In order to study the function of FvBck1, we first generated deletion mutants by replacing *FvBCK1* gene with hygromycin phosphotransferase (*HPH*) gene as the selectable marker (Supplementary Image 1A) using a standard transformation strategy (Hou et al., 2002). We obtained two independent deletion mutants (ΔFvbck1 and ΔFvbck1-8) after screening transformants by PCR. Subsequently, we performed gene complementation by reintroducing the wild-type *FvBCK1* gene, which was amplified along with its native promoter (Supplementary Image 1A), into the protoplast of ΔFvbck1 strain. We confirmed the gene replacement in ΔFvbck1 and ΔFvbck1-8 and the gene complementation in FvBck1-C and FvBck1-C2 by Southern blot and RT-PCR analyses (Supplementary Images 1B,C). Deletion mutants ΔFvbck1 and ΔFvbck1-8 shared same phenotypes, thus we selected ΔFvbck1 strain for further functional characterization.

### FvBck1 is important for hyphal growth and conidiation

**Figure 1:**
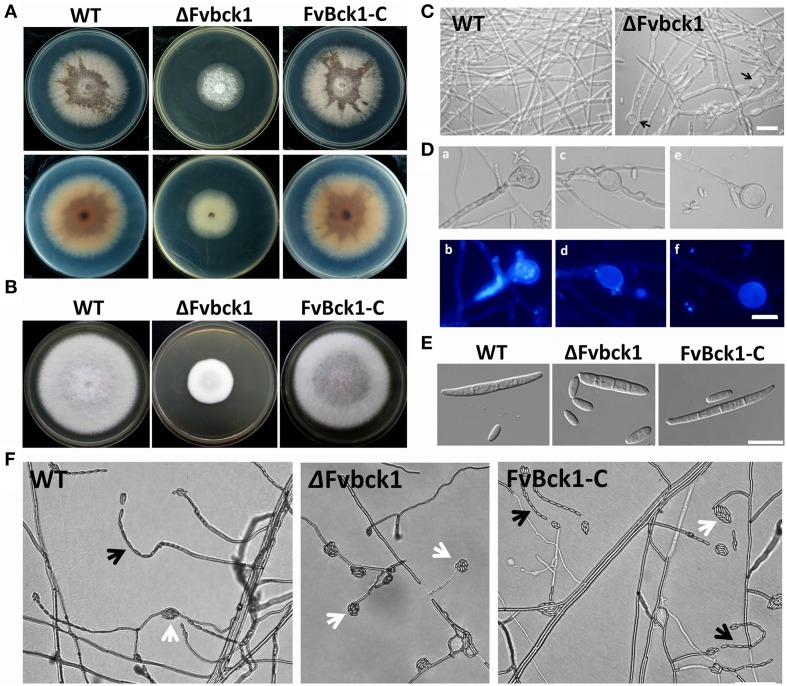
**Hyphal growth and conidiation phenotypes of wild-type (WT), FvBck1 deletion mutant (ΔFvbck1), and complementary (FvBck1-C) strains. (A)** Strains grown on SJA plates for 5 days. Upper panels are top view, and lower panels are bottom view. **(B)** Strains grown on CM plates for 5 days. **(C)** Hyphal morphology of WT and ΔFvbck1 strains grown in liquid CM medium for 3 days. The black arrows indicate swollen structures in the hyphae of ΔFvbck1 strain. Bar = 50 μm. **(D)** Detailed view of swollen structures in ΔFvbck1 hyphae. Hyphae were stained with 10 μg/ml 4′,6-diamidino-2-phenylindole (DAPI) and examined under differential interference contrast (DIC) **(a,c,e)** microscopy or UV light **(b,d,f)**. Bar = 20 μm. **(E)** Conidiation of each strain grown in MBJ medium for 7 days under white and UV light. Bar = 20 μm. **(F)** Microconidiation of each strain grown on solid modified Bilai's medium for 5 days. The black and white arrows indicate microconidial chain and false heads, respectively. Bar = 50 μm.

*FvBCK1* deletion mutant (ΔFvbck1), the complement (FvBck1-C), and the wild-type (WT) strains were grown on sugarcane juice agar (SJA) and complete medium (CM) broth for 5 days. The deletion mutant grew significantly slower than WT and complementation strains (Figures 1A,B), and did not produce brown pigment on SJA (Figure 1A). In CM broth all strains grew well, however after 3 days some swollen structures were observed both at the tip and in the middle of hyphae in ΔFvbck1 strain (Figures 1C,D). In the mutant, we observed multiple nuclei in about 10% of the swollen structures when stained with 4′,6-diamidino-2-phenylindole (DAPI) (Figures 1Da,b), and enlarged vacuole was observed in the majority of other cells (Figures 1Dc–f).

In mung bean juice (MBJ) culture, ΔFvbck1 produced about 70% less macroconidia compared to the WT after 7 days while no significant difference was observed in microconidia production. Macroconidia of ΔFvbck1 were shorter in length and slightly wider in width exhibiting a more stubby appearance when compared to those observed in WT and FvBck1-C (Figure 1E). WT and FvBck1-C strains produced microconidia in both microconidial chain and false-head forms on modified Bilai's medium, while ΔFvbck1 strain only produced the false-head microconidia (Figure 1F).

### FvBck1 plays a conserved role in cell wall integrity pathway

**Figure 2:**
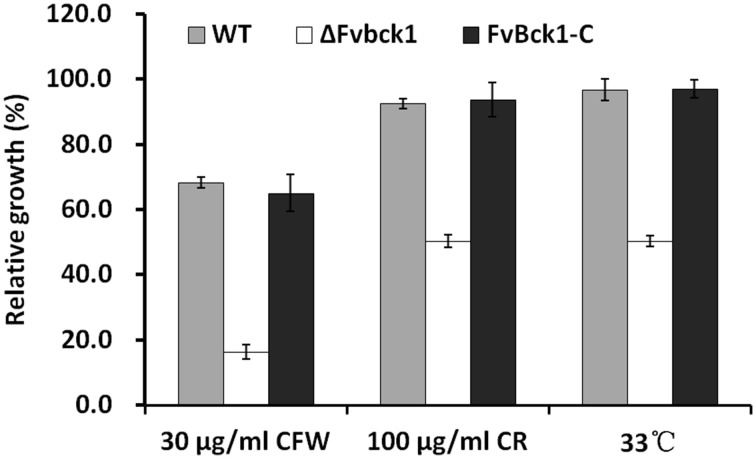
**The sensitivity of WT, ΔFvbck1, and FvBck1-C strains to cell wall damaging agents and high temperature**. The relative growth of each strain grown on CM plates with Calcofluor white (CFW) or Congo red (CR) at 26°C or without anything at 33°C for 4 days.

The Slt2/Mpk1 MAPK signaling pathway has been known to regulate cell wall integrity in fungi, including in *S. cerevisiae* and *M. oryzae* (Xu, 2000; Zhao et al., 2007; Li et al., 2012). Bck1 is the most upstream element known in the cell wall integrity MAPK pathway (Xu, 2000; Zhao et al., 2007), and we predicted that the disruption of FvBck1 negatively impacts the *F. verticillioides* cell wall integrity. As anticipated, the ΔFvbck1 mutant displayed an elevated level of sensitivity to cell wall disrupting agents, e.g., Calcofluor white (CFW) or CR, when compared to WT and FvBck1-C, indicating a conserved function of FvBck1 in cell wall integrity (Figure 2). Interestingly, ΔFvbck1 was also more sensitive to higher temperature (33°C) than WT and FvBck1-C (Figure 2), leading us to hypothesize that FvBck1 is involved in membrane fluidity homeostasis similar to that observed in *S. cerevisiae* (Lockshon et al., 2012).

### FvBck1 is required for virulence to host and FB1 production

**Figure 3:**
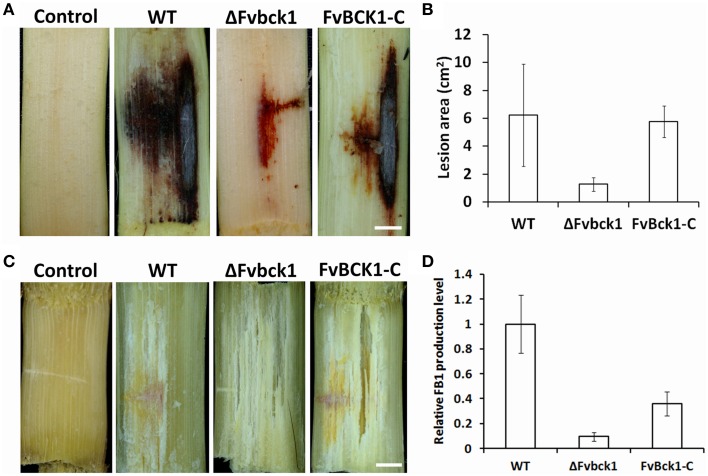
**Infection assays and FB1 production of WT, ΔFvbck1, and FvBck1-C strains**. **(A)** Disease symptom of sugarcane stems infected with 10^6^ conidia of each strain for 7 days. Bar = 1 cm. **(B)** Lesion area of longitudinally dissected sugarcane stalk infected with each strain for 7 days. **(C)** Hyphal growth observed in the autoclaved sugarcane stems inoculated with 10^6^ conidia of each strain for 7 days. Bar = 1 cm. **(D)** Relative FB1 production of each strain grown in autoclaved corn kernels for 7 days. FB1 levels were analyzed after normalization, and the average production level of WT strain was set to 1.

To test whether the deletion of *FvBCK1* influences the virulence in *F. verticillioides* we inoculated conidia (10^6^) of WT, ΔFvbck1, and FvBck1-C strains into sugarcane stalks. The result showed a significantly reduced rot symptom in ΔFvbck1 when compared to WT and FvBck1-C strains (Figure 3A and Supplementary Image 4A). Subsequently, we reisolated these fungal strains from the infected sugarcanes and incubated them on SJA medium to verify that symptoms on sugarcanes were caused by corresponding *F. verticillioides* strains (Supplementary Image 5). When we quantified the lesion areas in sugarcane stalks, ΔFvbck1 strain showed 1.5 cm^2^ of average symptom area compared to 6 cm^2^ in WT and FvBck1-C strains (Figure 3B). As a comparison, we also infected autoclaved sugarcane stalks with 10^6^ conidia of WT, ΔFvbck1, and FvBck1-C strains. After 7 days of incubation, we confirmed that all three strains grow equally well in the autoclaved sugarcanes, indicating that decreased virulence in ΔFvbck1 was not solely due to the growth defect observed on synthetic media (Figure 3C and Supplementary Image 4B). We also performed similar infection assays on corn stalks, and obtained similar results (Supplementary Image 6). *F. verticillioides* is known to produce mycotoxin FB1 on corn kernels, and we tested the levels of FB1 by inoculating and inducing kernel rot with these strains. The result showed the ΔFvbck1 strain produced significantly less FB1 than WT and FvBck1-C strains (Figure 3D), suggesting that FvBck1 also influences FB1 production.

### Addition of osmoregulators partially restored growth defects in ΔFvbck1 strain

**Figure 4:**
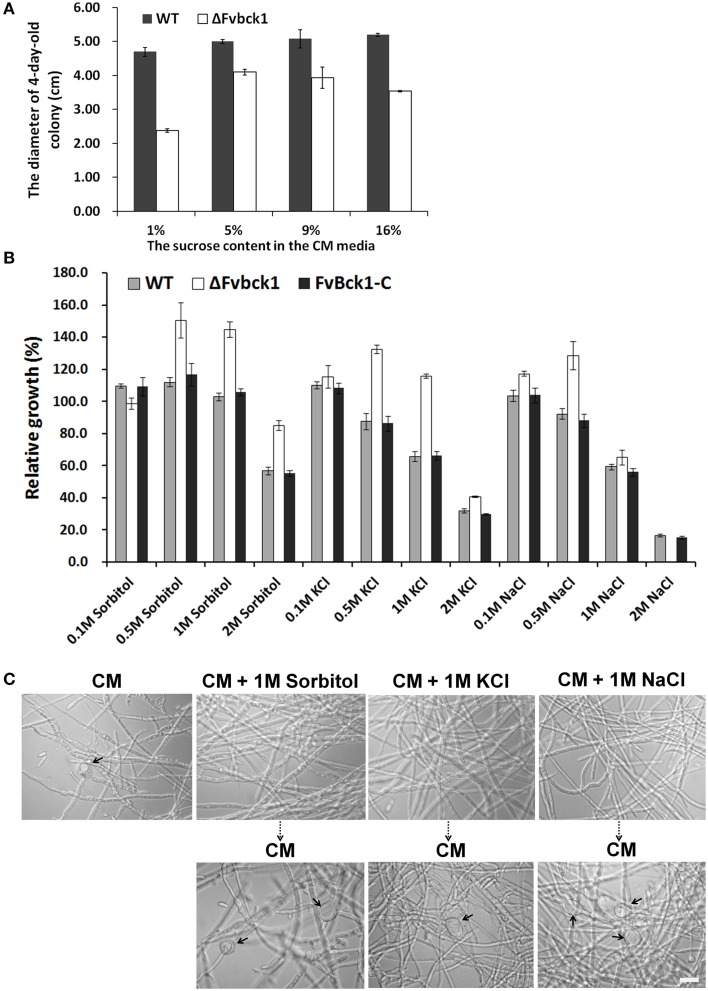
**The sensitivity of WT, ΔFvbck1, and FvBck1-C strains to osmotic stress**. **(A)** The colony diameter of WT and ΔFvbck1 strains grown on CM plates with different concentration of sucrose for 4 days. **(B)** Relative growth of WT, ΔFvbck1, and FvBck1-C strains grown on CM plates with different concentration of Sorbitol or KCl or NaCl for 4 days. Strains grown on CM plates considered as control (100%). **(C)** Hyphal morphology of ΔFvbck1 strain grown in liquid CM medium with or without 1 M Sorbitol or KCl or NaCl for 3 days (upper panels) and then shift to CM medium for an additional day (lower panels). The arrows indicate the swollen structures. Bar = 20 μm.

The mutant (ΔFvbck1) showed growth defect on SJA and CM agar plates but not in autoclaved sugarcanes. Considering that the sucrose content in sugarcane juice is about 11 ~ 16% (w/v) (Tang, 2012), we amended standard CM agar (normally 1% sucrose) with higher levels of sucrose, e.g., 5, 9, and 16%, to test whether higher levels of sucrose can complement ΔFvbck1 growth. Our results demonstrated that the ΔFvbck1 strain grew better on CM agar with 5, 9, or 16% sucrose than on standard CM agar (*P* < 0.05), while no significant difference was observed when the WT strain was grown on CM plates with different sucrose levels (Figure 4A).

Subsequently, we incubated WT, ΔFvbck1, and FvBck1-C strains on CM agar amended with different concentrations of sorbitol, KCl and NaCl. After 5 days, the relative growth of these strains on different media revealed that the adequate concentration of sorbitol, KCl or NaCl in CM agar can facilitate vegetative growth in ΔFvbck1. In contrast, we observed no significant difference of vegetative growth in WT and FvBck1-C on sorbitol (0.5 or 1 M) but rather inhibitory effect on KCl (0.5 or 1 M) and NaCl (0.5 M) (Figure 4B). Our results suggested that ΔFvbck1 exhibits better adaptation to higher concentration of sucrose, sorbitol, KCl, and NaCl, which contributes to partial restoration of vegetative growth.

As mentioned earlier, ΔFvbck1 produces swollen structures in hyphae when grown in CM broth. We incubated the ΔFvbck1 strain in the CM broth containing 1 M sorbitol, KCl, and NaCl for 3 days to test if the addition of osmoregulators could also remove swollen hyphal structures. No swollen structure was observed when the mutant was grown in these cultures but they reemerged after just 1 day when we shifted the hyphae from these cultures to standard CM broth (Figure 4C).

### FvBck1 regulates catalase and peroxidase expression

**Figure 5:**
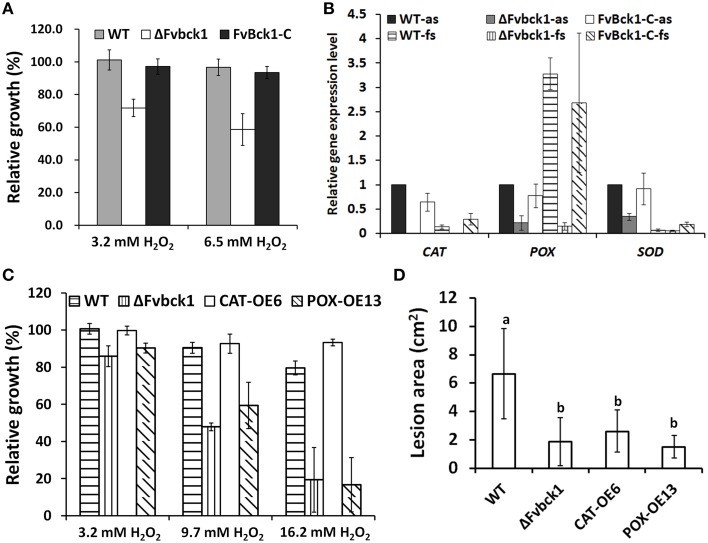
**The sensitivity to H_2_O_2_ and virulence of WT, ΔFvbck1, FvBck1-C, CAT-OE6, and POX-OE13 strains. (A)** Relative growth of WT, ΔFvbck1, and FvBck1-C strains grown on CM plates with different concentration of H_2_O_2_ for 4 days. **(B)** Relative gene expression level of *CAT* (FV12888), *POX* (FV11221), and *SOD* (FV01940) of WT, ΔFvbck1, and FvBck1-C strains grown in autoclaved or fresh sugarcanes for 7 days. The expression level of WT strain grown in autoclaved sugarcane was set to 1. as, autoclaved sugarcane; fs, fresh sugarcane. **(C)** The relative growth of WT, ΔFvbck1, CAT-OE6, and POX-OE13 strains grown on CM plates with different concentration of H_2_O_2_ for 3 days. **(D)** Lesion area of each rip cut sugarcane stem infected by each strain for 7 days. Mean and standard error were calculated from three independent biological replicates. Different letters were used to mark statistically significant difference (*P* < 0.05).

The mutant strain is less virulent when inoculated into sugarcane stalks, and we questioned whether the mutant was more sensitive to stress agents secreted by the host during colonization. For instance, oxidative burst often occurs *in planta* when infected with pathogens, and hydrogen peroxide (H_2_O_2_) is one of the well-known reactive oxidative species (ROS) produced during host-pathogen interactions (Mehdy, 1994; Low and Merida, 1996). WT, ΔFvbck1, and FvBck1-C strains were grown on CM plates with or without H_2_O_2_ for 4 days, and the result showed that the ΔFvbck1 strain was more sensitive to H_2_O_2_ than WT and Fvbck1-C strains (Figure 5A).

As for the H_2_O_2_ sensitivity observed in ΔFvbck1 strain, we hypothesized that the capacity of eliminating ROS in the ΔFvbck1 strain is negatively affected. In *F. graminearum*, three putative extracellular H_2_O_2_ or ${\text{O}}_{2}^{-}$ scavenging enzymes, i.e., FGSG_12369 (a putative catalase), FGSG_04434 (a putative ascorbate peroxidase), and FGSG_08721 (a putative superoxide dismutase), were significantly up-regulated when *F. graminearum* was grown inside wheat coleoptiles when compared to *in vitro* samples (Zhang et al., 2012). We identified three gene encoding these enzymes in *F. verticillioides*: FVEG_12888, FVEG_11221, and FVEG_01940. We further tested the relative expression of these genes in WT, ΔFvbck1, and FvBck1-C strains when cultured for 7 days in fresh sugarcane vs. autoclaved sugarcane. The result showed that all three genes in ΔFvbck1 were significantly down-regulated, particularly the catalase gene (FVEG_12888, designated *CAT*) which was almost not detectable in both autoclaved and fresh sugarcanes when compare to the WT and FvBck1-C (Figure 5B). Moreover, we found that the peroxidase gene (FVEG_11221, designated *POX*) in WT or Fvbck1-C strains was up-regulated dramatically, although the other two genes were down-regulated in fresh sugarcane compared to autoclaved sugarcane (Figure 5B).

To further investigate whether catalase and peroxidase are associated with reduced virulence observed in the mutant, we independently overexpressed *CAT* and *POX* genes in the ΔFvbck1 strain (Supplementary Image 7). We selected CAT-OE6 and POX-OE13 strains for sensitivity test to H_2_O_2_, and the result showed that CAT-OE6 exhibited a WT-like response to H_2_O_2_ whereas POX-OE13 was more aligned with ΔFvbck1 (Figure 5C). However, both CAT-OE6 and POX-OE13 strains failed to recover virulence even when sugarcane stalks were monitored for 7 days after inoculation (Figure 5D and Supplementary Image 8).

### Partial complementation of the *FvBck1* deletion mutant by *M. oryzae MCK1*

**Figure 6:**
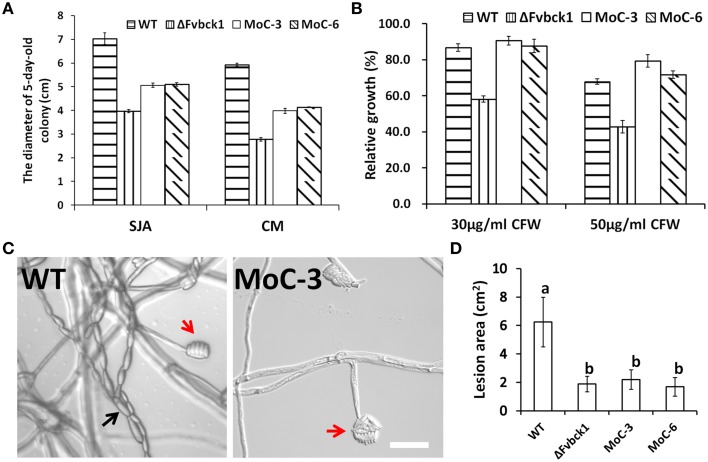
**Mck1 partially restored the defects of ΔFvbck1. (A)** Strains grown on SJA or CM plates for 5 days. **(B)** Relative growth of each strain grown on CM plates with different concentration of CFW at 26°C for 3 days. **(C)** Microconidiation of each strain grown on SJA plates for 5 days. Black and Red arrows indicate microconidial chain and false heads, respectively. Bar = 50 μm. **(D)** Lesion area of longitudinally dissected sugarcane stalks infected by each strain for 7 days. Mean and standard error were calculated from three independent biological replicates. Different letters were used to mark statistically significant difference (*P* < 0.05).

To test if *MCK1*, the Bck1 homolog in *M. oryzae*, can functionally complement the *FvBCK1* mutant, we transformed *MCK1* gene under the control of its native promoter together with pKNT vector, which contains a neomycin gene, into the ΔFvbck1 strain. We screened transformants by PCR and RT-PCR to confirm the expression of *MCK1*. Of the transformed isolates we selected two, MoC-3 and MoC-6, for further analysis. For vegetative growth, we found that these two strains grew faster than ΔFvbck1 but still slower than the WT on CM and SJA plates (Figure 6A), and that these strains only partially recovered the capacity of pigment production on SJA plates (Supplementary Image 9). While MoC-3 and MoC-6 showed complete restoration of sensitivity to calcofluor white (Figure 6B), these strains failed to exhibit WT level of conidiation and virulence (Figures 6C,D).

## Discussion

In this study, we investigated the role of MAPK signaling pathways in *F. verticillioides* virulence associated with sugarcane Pokkah Boeng disease. The FvBck1 we identified shares high homology with *S. cerevisiae* Bck1 and *M. oryzae* Mck1 and possess all the conserved domains attributed to this group of proteins. Our premise was that FvBck1 plays roles similar to the orthologs in *S. cerevisiae* and *M. oryzae*, which are MAPKK kinase proteins that participate in the cell wall integrity pathway (Slt2/Mpk1pathway) and regulate many physiological functions including vegetative growth, virulence, conidiation, response to osmotic stress, and FB1 production (Park et al., 2008; Valiante et al., 2008, 2009; Wang et al., 2011; Li et al., 2012).

We further observed that, the deletion of *FvBCK1* (ΔFvbck1) resulted in reduced radial growth of ΔFvbck1 mutants on SJA and CM plates (Figures 1A,B). This result shows that *FvBCK1* is involved in the promotion of vegetative growth and further confirmed previous findings that implicated *BCK1* in the growth of *N. crassa, A. fumigatus* as well as *F. graminearum* (Park et al., 2008; Valiante et al., 2009; Wang et al., 2011). More so, the growth defects exhibited by ΔFvbck1 mutants sharply contradict growth characteristics displayed by *M. oryzae MCK1* deletion mutants which portrayed normal radial growth but was associated with autolysis of mycelia when the strain was incubated on oatmeal agar plate (Jeon et al., 2008). The morphological variation exhibited by ΔFvbck1 mutants compared with *MCK1* mutants suggests that *F. verticillioides FvBCK1* performs growth related functions similar to *N. crassa, A. fumigatus*, and *F. graminearum*. Interestingly, the characteristic defect in radial growth of ΔFvbck1 mutants was restored to the wild-type level upon subsequent supplementation of growth mediums with osmoregulators, i.e., sorbitol, KCl, and NaCl (Figure 4B). These observations coupled with evidence from previous investigations, e.g., the application of 1 M sorbitol was enough to aid *A. fumigatus* Δbck1 mutant to overcome sensitivity to glucanex, a compound with high chitinase and glucanase activity (Valiante et al., 2009), justify our conclusion that Bck1 performs crucial role in promoting hyphae growth in *F. verticillioides*. It also mediates the regulation of cell wall integrity in *F. verticillioides* but in a manner diverse from other fungal species.

Slt2/Mpk1 pathway is known to be essential for cell wall integrity in fungi, and *F. verticillioides* is no exception (Zhao et al., 2007; Li et al., 2012); the ΔFvbck1 strain is not only sensitive to cell wall damaging agents but also to elevated temperature (33°C) (Figure 2). In *F. graminearum*, the deletion of *MGV1*, a Slt2 homolog, resulted in sensitivity to driselase and elevated incubation temperatures (32°C) (Hou et al., 2002). Furthermore, *F. graminearum* mutant generated swollen bodies when incubated at 32°C (Hou et al., 2002). Our study showed ΔFvbck1 strain generated swollen structures in CM liquid culture at normal incubation temperature (26°C) and these structural abnormalities can be rescued with appropriate osmotic stress (Figure 4B). These results suggest that Slt2/Mpk1 pathway of the two *Fusarium* species influences cell wall integrity and osmotic pressure balance.

In addition to hyphal growth, FvBck1 is important for both microconidia and macroconidia production. Conidiation was drastically reduced in *M. oryzae MCK1* deletion strain and *F. graminearum* Δbck1 strain (Jeon et al., 2008; Wang et al., 2011). In *F. verticillioides*, macroconidia production was reduced significantly in the mutant, but microconidia production was similar to the WT strain. However, macroconidia produced by ΔFvbck1 mutants lacked the typical microconidial chains (Figure 1F). The conidiation defects displayed by ΔFvbck1 mutants are similar to those observed in adenylate cyclase deletion mutant Δfac1, which lacked macroconidia and only produced microconidia on false heads (Choi and Xu, 2010). These findings suggest that the Slt2/Mpk1 and cAMP pathways may be associated with closely related downstream targets to co-regulate conidation, especially the formation microconidia chain in *F. verticillioides*.

Unlike the cAMP signaling pathway, which has minimal, if any, involvement in FB1 biosynthesis (Choi and Xu, 2010), MAPK signaling pathways seem to play important roles in FB1 production. The ΔFvbck1 strain produces much less FB1 than the WT strain, suggesting that a functional CWI MAPK pathway is necessary for proper FB1 biosynthesis (Figure 3D). This outcome is similar to the phenotype observed with *FvMK1* mutation, another MAPK in *F. verticillioides* (Zhang et al., 2011). Interestingly, FB1 production of the complementary strain was restored but not completely when compared to WT and ΔFvbck1 strains. It is possible that the random insertion of *FvBCK1* ORF in the complementary strain might have affected the normal expression level of *FvBCK1* gene and the FB1 production. Moreover, the ΔFvbck1 strain did not produce pigments on SJA plate suggesting that FvBck1 is also involved in the regulation of pigment production in *F. verticillioides* (Figure 1A). In *N. crassa*, melanin accumulation is affected when the MAK-1 (Slt2) MAPK cascade was disrupted (Park et al., 2008). In contrast, functional MpkA (Slt2) MAPK cascade inhibits the pyomelanin formation in *A. fumigatus* (Valiante et al., 2009). These suggest that the Slt2 MAPK cascade plays specie-specific role in the metabolism of fungal secondary metabolites.

MAPK signaling pathways have been well characterized and are known to be critical for pathogenicity in *M. oryzae* and *F. graminearum* resulting in rice blast and wheat scab, respectively (Ramamoorthy et al., 2007; Wang et al., 2011; Li et al., 2012; Zheng et al., 2012). In addition, FvMK1 was shown to be critical for virulence of *F. verticilliodes*, on corn (Zhang et al., 2011). Further investigation of the ΔFvbck1 mutants generated in our study shows that the deletion of FvBck1 significantly impedes the virulence of *F. verticilliodes* on both sugarcane and corn (Figures 3A,B and Supplementary Image 6). And in view of the fact that the mutation of FvBck1 reduced vegetative growth in ΔFvbck1 mutants on synthetic media (Figures 1A,B), it was prudent for us to conclude that the defective growth constituted the prime reason for reduction in virulence. However, adequate addition of osmoregulators restored the growth defect in ΔFvbck1 strain (Figure 4). Furthermore, the mutant grew as well as the WT in autoclaved sugarcane, which contains a higher level of sucrose (Figure 3C), and thereby indicating that the growth defect associated with the mutation may not be the sole factor accounting for reduced virulence. We therefore reasoned that host resistance mechanisms, e.g., oxidative burst, could be one of the possible factors responsible for reduced virulence in ΔFvbck1 mutants.

We observed that putative peroxidase gene (*POX*) was up-regulated when WT strain was cultured on fresh sugarcane compare to autoclaved sugarcane, suggesting the occurrence of an oxidative burst in a living host when inoculated with *F. verticillioides* (Figure 5B). Furthermore, three genes that encode putative extracellular H_2_O_2_ or ${\text{O}}_{2}^{-}$ scavenging enzymes were significantly down regulated in ΔFvbck1 mutants, and these observations subsequently provided clues for proposing one possible mechanism responsible for the high sensitive to oxidative stress (Figures 5A,B). These results further show that the deletion of FvBck1 impaired the ability of ΔFvbck1 mutant to defend itself against ROS generated by the host and provide one possible argument for the reduced virulence associated with the ΔFvbck1 mutants. However, virulence was not completely restored when we overexpressed these genes in the mutant, we therefore hyposthesize that other yet-to-be determined physiological factors might have also contributed the reduction in virulence of ΔFvbck1 mutants.

The Slt2/Mpk1 pathway regulates several transcription factors (TFs) in *S. cerevisiae*, such as Rlm1, Swi4, and Swi6 (Zhao et al., 2007). Rlm1 is a MADS-box TF, and a recent study revealed that a Rlm1 homolog in *F. verticillioides* FvMads2 contributes to cell wall integrity but is dispensable for pathogenicity (Ortiz and Shim, 2013). Thus, we hypothesized that another TF regulated by Slt2/Mpk1 pathway might perhaps be responsible for pathogenicity. In *F. graminearum*, FgSwi6 is involved in virulence to wheat (Liu et al., 2013), and we further need to test whether a homolog of FgSwi6 performs similar function in *F. verticillioides*. We also identified a gene (FVEG_12888), which encodes a putative catalase, whose expression was not detectable in ΔFvbck1 (Figure 5B). We can also hypothesize that the expression of this gene is controlled by a TF downstream of Slt2/Mpk1pathway, but further examination is warranted.

Heterologous complementation of *M. oryzae MCK1* into ΔFvbck1 mutant failed to completely restore conidiation and virulence, albeit the fact that *MCK1* and *FvBCK1* are important for virulence in *M. oryzae* and *F. verticillioides*, respectively (Figures 6C,D). This result suggests that these two fungi have different downstream regulatory mechanism for virulence and conidiation. While Bck1 homologs are known to play a conserved role in cell wall integrity (Figure 6B), our study, along with studies performed in other organisms, provides reasonable evidence that Bck1 performs species-specific functions that includes virulence and reproduction in addition to cell wall integrity. We can also reason that our heterologous complementation strategy failed to restore certain phenotypic deficiencies due to gene specific cis- and trans-regulatory elements associated with FvBck1 function. Future efforts aimed at investigating the genetic association between Slt2/Mpk1 pathway and the putative downstream TFs would greatly enhance the understanding of how *F. verticillioides* regulates Pokkah Boeng on sugarcane.

## Author contributions

Conceived and designed the experiments: CZ, GL, WS, ZW. Performed the experiments: CZ, JW, HT, XD, YW, MC, ZZ, WY. Analyzed the data: CZ, LX, GL, WS, ZW. Wrote the paper: CZ, LX, WS, ZW. Originated research leading up to this paper and provided guidance and review: LX, GL, WS, ZW.

## Funding

This work was supported by National Natural Science Foundation, China (31030004), the earmarked fund for the Modern Agriculture Technology of China (CARS-20) and the Scientific Research Foundation of Graduate School of Fujian Agriculture and Forestry University.

### Conflict of interest statement

The authors declare that the research was conducted in the absence of any commercial or financial relationships that could be construed as a potential conflict of interest.

## Supplementary material

Supplementary Image 1 **Generation of *FvBCK1* deletion mutant and complementary strain. (A)** *FvBCK1* gene locus and gene replacement construct. The *FvBCK1* and *hph* gene are marked with empty and black arrow, respectively. K, *Kpn* I. **(B)** DNA gel blots of restriction enzymes marked in **(A)** digested genomic DNA of WT, ΔFvbck1 and FvBck-C strains were hybridized with probe amplified with primer pair PF/PR marked in **(A)**. **(C)** Total RNA samples isolated from mycelia of WT, ΔFvbck1, ΔFvbck1-8, FvBck-C, and FvBck-C2 strains were subjected to RT-PCR using *FvBCK1* gene-specific primers QF-BCK1/QR-BCK1 (Table S1), *ACTIN* gene (FVEG_03116) was amplified using QF-ACT/QR-ACT primers as positive control.

Supplementary Image 2 **Sugarcane stalk inoculation assay. (A)** Schematic description of the inoculation strategy. **(B)** Preliminary stalk rot assay with different amount of WT conidia suspension.

Supplementary Image 3 **Phylogenic analysis of components of MAP Kinase signaling pathways in yeast and filamentous fungi**. The phylogenetic tree compiled from the amino acid sequences was drawn using Clustal X and MAGA3.1 softwares. The phylogenetic analysis was performed using ClustalX based on amino acid sequences from *Saccharomyces cerevisiae* (Scbck1, ScSte11, ScSsk2, ScSsk22, ScMkk1, ScMkk2, ScSte7, ScPbs2, ScHog1, ScSlt2, and ScFus3), *Aspergillus nidulans* (Anbck1, AnSte11, AnSSkB, AnMkk1, AnSte7, AnPbsB, AnHog1, AnSlt2, and AnPmk1), *Neurospora crassa* (NcMik-1, NcNrc-1, NcOs-4, NcMek-1, NcMek-2, NcOs-5, NcOs-2, NcMak-1, and NcMak-2), *Magnapothe oryzae* (MoMck1, MoMst11, MoSsk2, MoMkk1, MoMst7, MoPbs2, MoHog1, MoMps1, and MoPmk1), *Fusarium graminearum* (FgBck1, FgSte11, FgSsk2, FgMkk1, FgSte7, FgPbs2, FgHog1, FgMgv1, and FgGpmk1), and *Fusarium verticillioides* (FvBck1, FvSte11, FvSsk2, FvMkk1, FvSte7, FvPbs2, FvHog1, FvSlt2, and FvPmk1). The bar indicates the evolutionary distance.

Supplementary Image 4 **Sugarcane stem infection assays of WT and ΔFvbck1 strains. (A)** Disease development process of fresh sugarcane stems infected with 10^6^ conidia of WT or ΔFvbck1 strain in 7 days. **(B)** Fungal growth process in autoclaved sugarcane stems incubated with 10^6^ conidia of WT or ΔFvbck1 strain in 7 days.

Supplementary Image 5 **WT and ΔFvbck1 strains from the infected sugarcane stems grown on SJA plates for 3 days**.

Supplementary Image 6 **Disease symptom of corn stalks infected with WT, ΔFvbck1, and FvBck1-C strains for 15 days**.

Supplementary Image 7 **Relative gene expression level of *CAT* (FVEG_12888) and *POX* (FVEG_11221) of WT, CAT-OE6, and POX-OE13 strains grown in CM liquid media for 3 days**. The expression level of WT strain grown in autoclaved sugarcane was set to 1. Mean and standard error were calculated from three independent biological replicates.

Supplementary Image 8 **Disease symptom of sugarcane stems infected with 10^6^ conidia of WT, ΔFvbck1, FvBck1-C, CAT-OE6, and POX-OE13 strains for 7 days**. Bar = 1 cm.

Supplementary Image 9 **Pigment formation of WT, ΔFvbck1, MoC-3, and MoC-6 strains grown on SJA plates for 5 days**.

Supplementary Table S1 **PCR primers used in this study**.
