# Supplementary figures and images for: FvBck1, a component of cell wall integrity MAP kinase pathway, is required for virulence and oxidative stress response in sugarcane Pokkah Boeng pathogen
Source: Front Microbiol. 2015 Oct 8;6:1096. doi: 10.3389/fmicb.2015.01096 (PMC4597114; doi:10.3389/fmicb.2015.01096)

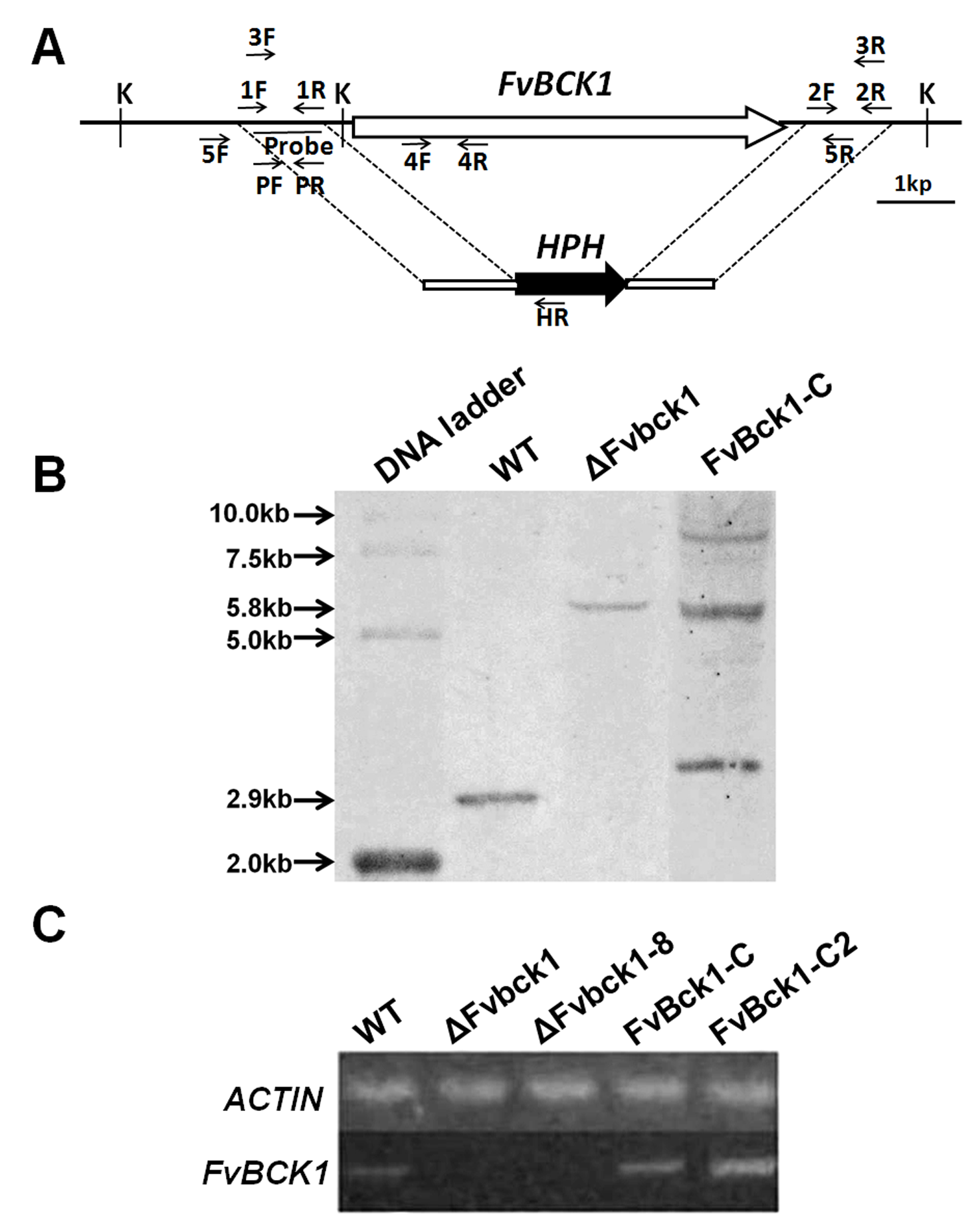

Supplement: Supplementary Image 1 — Generation of FvBCK1 deletion mutant and complementary strain. (A) FvBCK1 gene locus and gene replacement construct. The FvBCK1 and hph gene are marked with empty and black arrow, respectively. K, Kpn I. (B) DNA gel blots of restriction enzymes marked in (A) digested genomic DNA of WT, ΔFvbck1 and FvBck-C strains were hybridized with probe amplified with primer pair PF/PR marked in (A). (C) Total RNA samples isolated from mycelia of WT, ΔFvbck1, ΔFvbck1-8, FvBck-C, and FvBck-C2 strains were subjected to RT-PCR using FvBCK1 gene-specific primers QF-BCK1/QR-BCK1 (Table S1), ACTIN gene (FVEG_03116) was amplified using QF-ACT/QR-ACT primers as positive control. [file Image1.TIF]

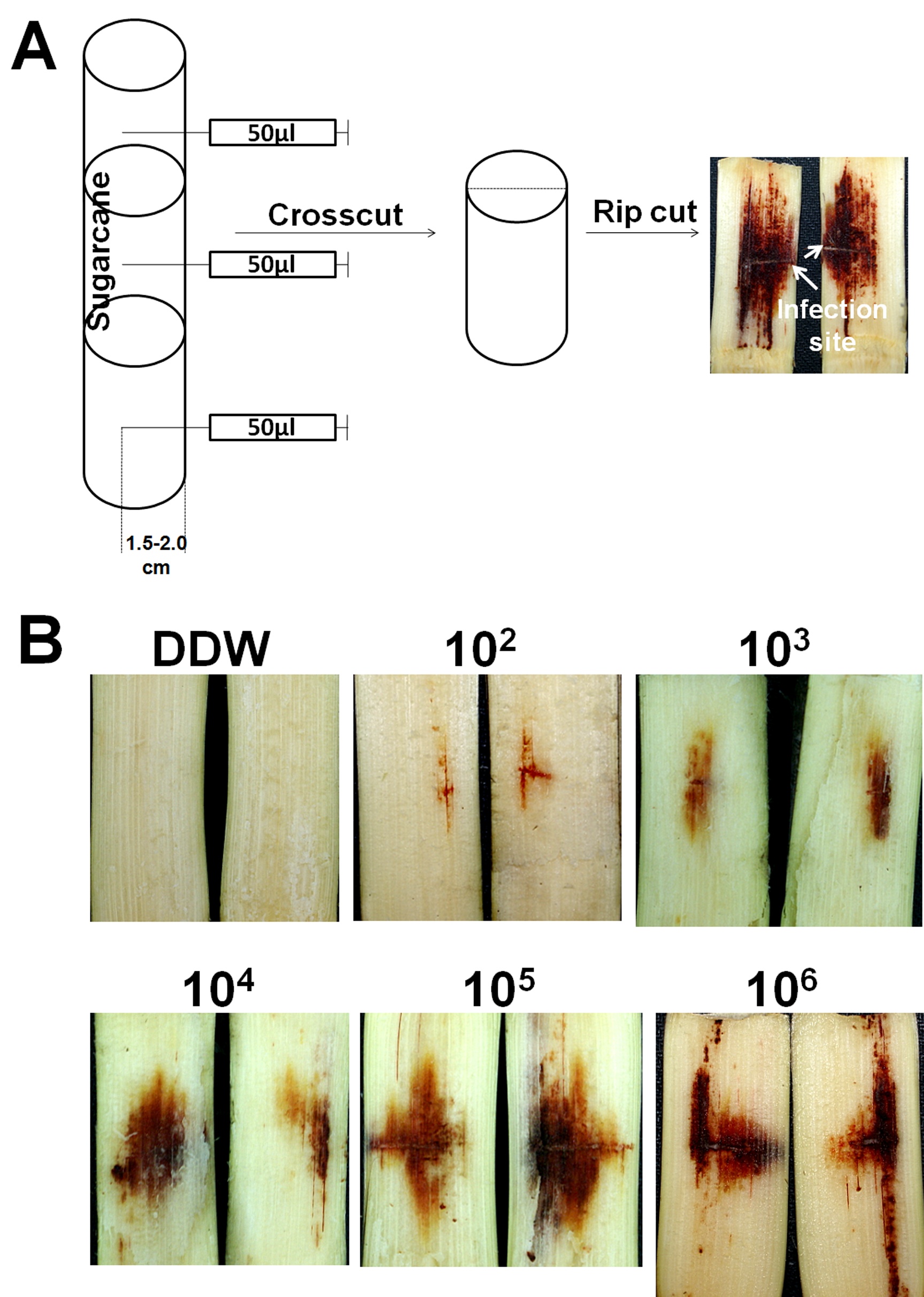

Supplement: Supplementary Image 2 — Sugarcane stalk inoculation assay. (A) Schematic description of the inoculation strategy. (B) Preliminary stalk rot assay with different amount of WT conidia suspension. [file Image2.TIF]

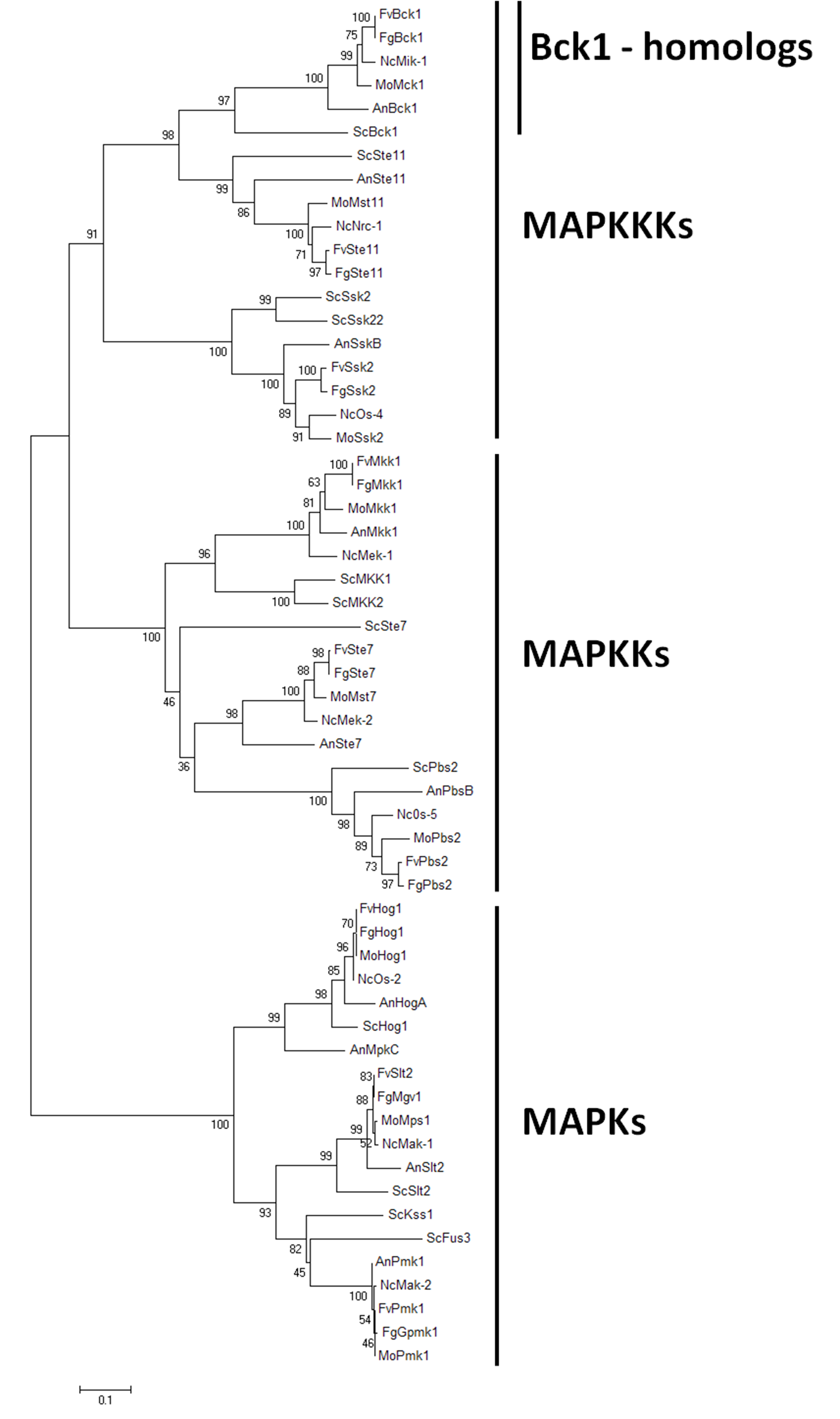

Supplement: Supplementary Image 3 — Phylogenic analysis of components of MAP Kinase signaling pathways in yeast and filamentous fungi. The phylogenetic tree compiled from the amino acid sequences was drawn using Clustal X and MAGA3.1 softwares. The phylogenetic analysis was performed using ClustalX based on amino acid sequences from Saccharomyces cerevisiae (Scbck1, ScSte11, ScSsk2, ScSsk22, ScMkk1, ScMkk2, ScSte7, ScPbs2, ScHog1, ScSlt2, and ScFus3), Aspergillus nidulans (Anbck1, AnSte11, AnSSkB, AnMkk1, AnSte7, AnPbsB, AnHog1, AnSlt2, and AnPmk1), Neurospora crassa (NcMik-1, NcNrc-1, NcOs-4, NcMek-1, NcMek-2, NcOs-5, NcOs-2, NcMak-1, and NcMak-2), Magnapothe oryzae (MoMck1, MoMst11, MoSsk2, MoMkk1, MoMst7, MoPbs2, MoHog1, MoMps1, and MoPmk1), Fusarium graminearum (FgBck1, FgSte11, FgSsk2, FgMkk1, FgSte7, FgPbs2, FgHog1, FgMgv1, and FgGpmk1), and Fusarium verticillioides (FvBck1, FvSte11, FvSsk2, FvMkk1, FvSte7, FvPbs2, FvHog1, FvSlt2, and FvPmk1). The bar indicates the evolutionary distance. [file Image3.TIF]

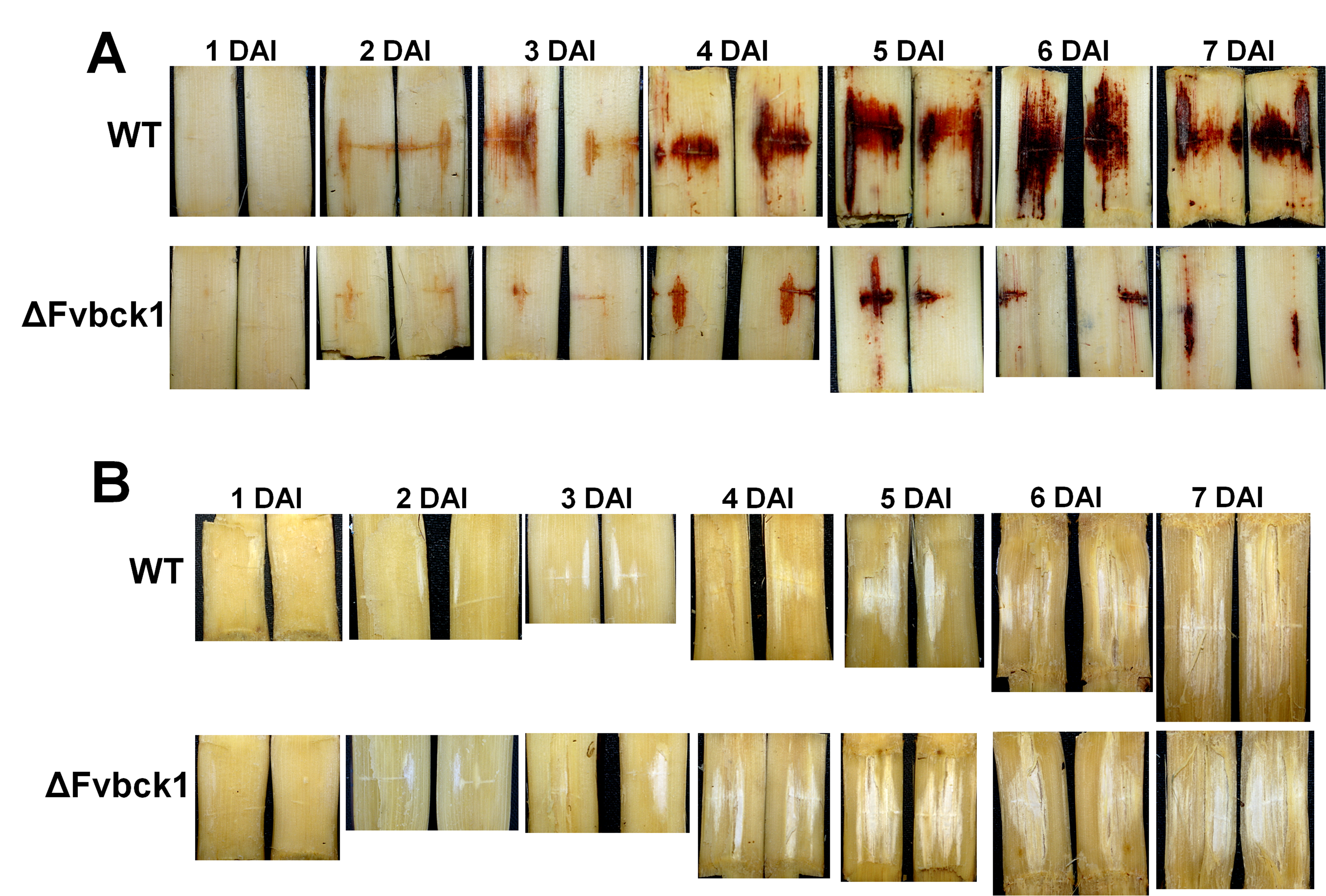

Supplement: Supplementary Image 4 — Sugarcane stem infection assays of WT and ΔFvbck1 strains. (A) Disease development process of fresh sugarcane stems infected with 106 conidia of WT or ΔFvbck1 strain in 7 days. (B) Fungal growth process in autoclaved sugarcane stems incubated with 106 conidia of WT or ΔFvbck1 strain in 7 days. [file Image4.TIF]

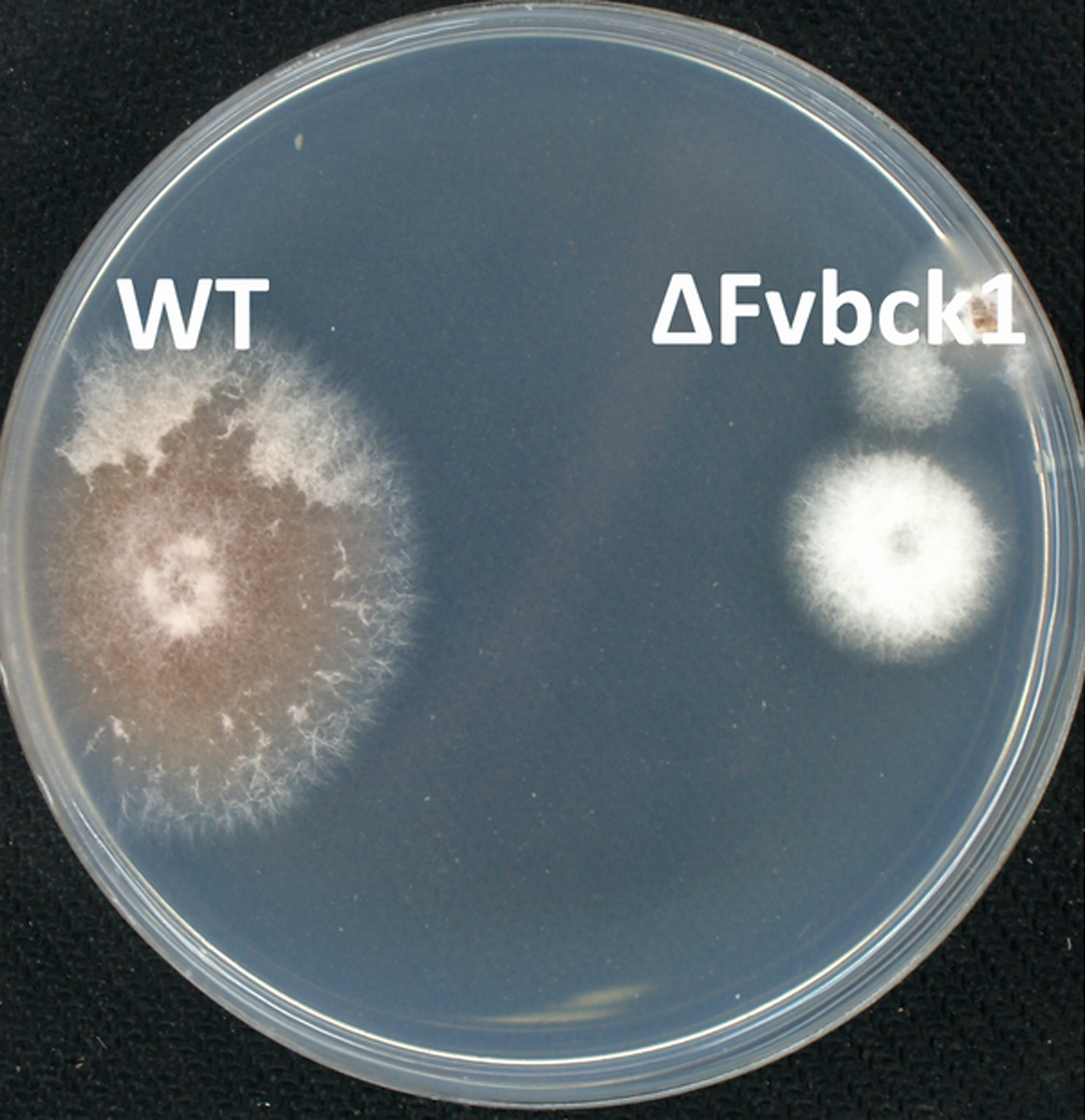

Supplement: Supplementary Image 5 — WT and ΔFvbck1 strains from the infected sugarcane stems grown on SJA plates for 3 days. [file Image5.TIF]

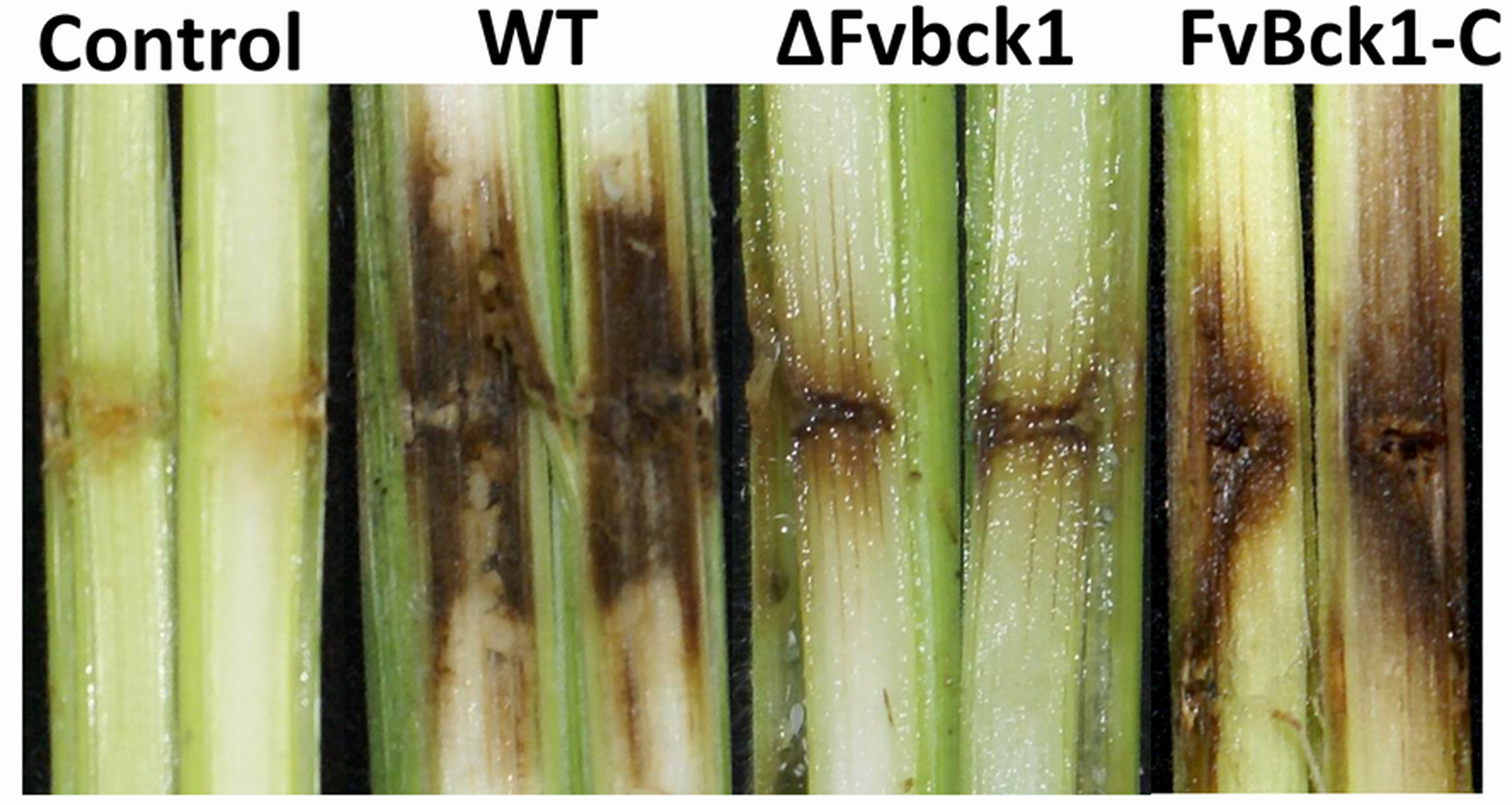

Supplement: Supplementary Image 6 — Disease symptom of corn stalks infected with WT, ΔFvbck1, and FvBck1-C strains for 15 days. [file Image6.TIF]

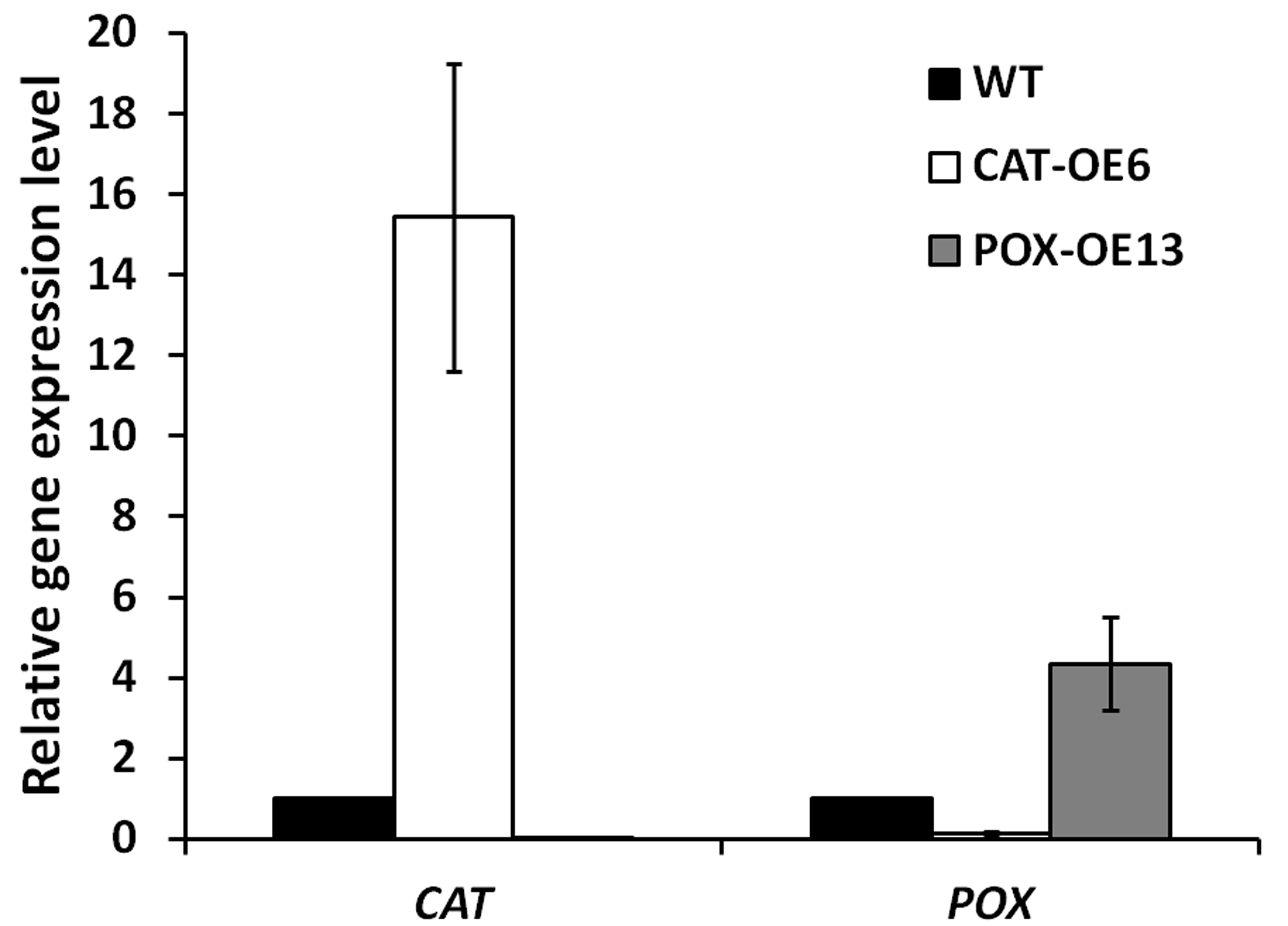

Supplement: Supplementary Image 7 — Relative gene expression level of CAT (FVEG_12888) and POX (FVEG_11221) of WT, CAT-OE6, and POX-OE13 strains grown in CM liquid media for 3 days. The expression level of WT strain grown in autoclaved sugarcane was set to 1. Mean and standard error were calculated from three independent biological replicates. [file Image7.TIF]

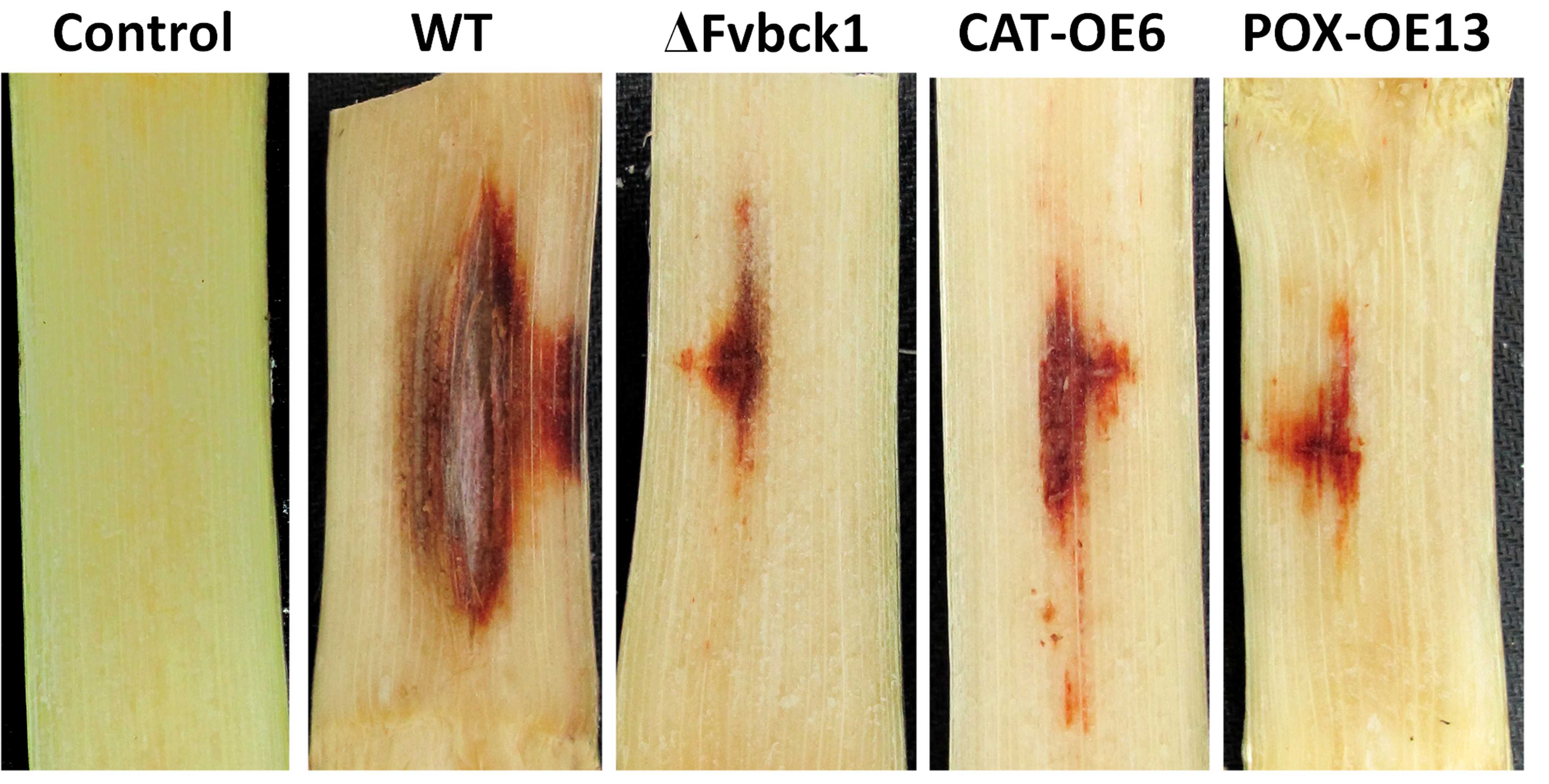

Supplement: Supplementary Image 8 — Disease symptom of sugarcane stems infected with 106 conidia of WT, ΔFvbck1, FvBck1-C, CAT-OE6, and POX-OE13 strains for 7 days. Bar = 1 cm. [file Image8.TIF]

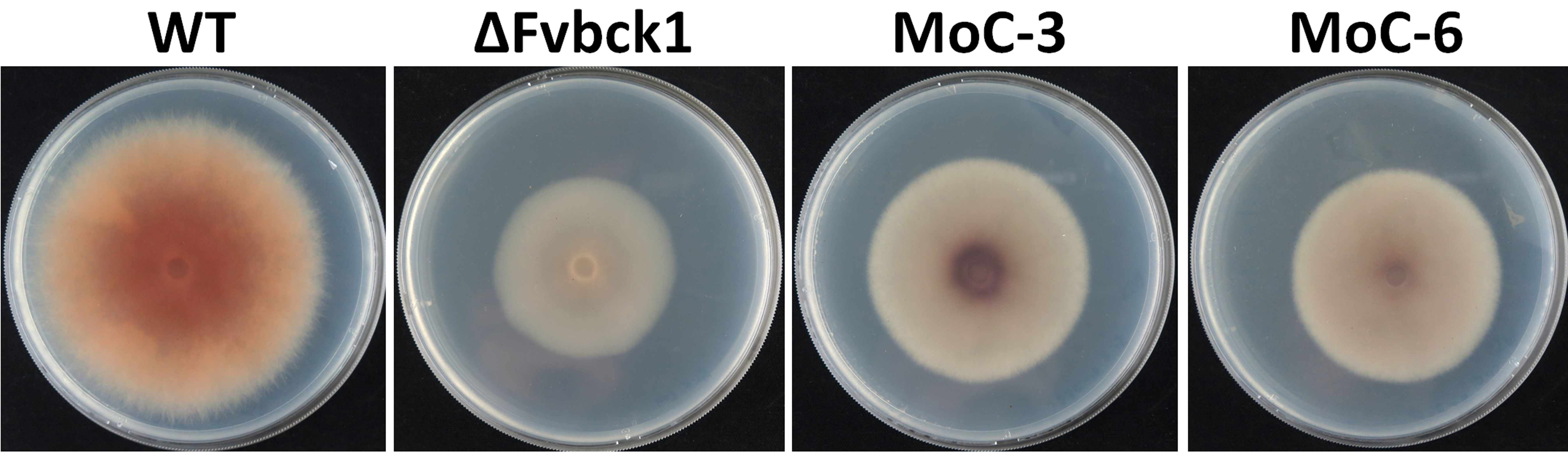

Supplement: Supplementary Image 9 — Pigment formation of WT, ΔFvbck1, MoC-3, and MoC-6 strains grown on SJA plates for 5 days. [file Image9.TIF]
